# Moving beyond the T cell synapse for combination neoadjuvant immunotherapy in head and neck cancer

**DOI:** 10.1172/JCI162733

**Published:** 2022-09-15

**Authors:** R. Bryan Bell, Michael Gough, Marka Crittenden, Kristina Young

**Affiliations:** Earle A. Chiles Research Institute, a Division of Providence Cancer Institute, Portland, Oregon, USA.

## Abstract

Patients with HPV-unrelated head and neck squamous cell carcinoma (HPV-unrelated HNSCC) show only modest benefit from treatment with PD-1 inhibitors (PD-1i). Targeting transforming growth factor β (TGF-β) may make PD-1i more effective by inducing T cell responses. In this issue of the *JCI*, Redman et al. performed a clinical trial in 14 patients with HPV-unrelated HNSCC using bintrafusp alfa, a bifunctional fusion protein that blocks PD-L1 and TGF-β. Primary tumors displayed pathologic responses with 5 of 14 patients having at least a partial response. While no primary tumor or metastatic lymph node demonstrated a complete pathologic response, the findings suggest that concurrent neoadjuvant inhibition of PD-L1 and TGF-β may provide a rational strategy to improve pathologic response and clinical outcome in patients with HPV-unrelated HNSCC.

## Tumors evade immune surveillance

**Figure 1:**
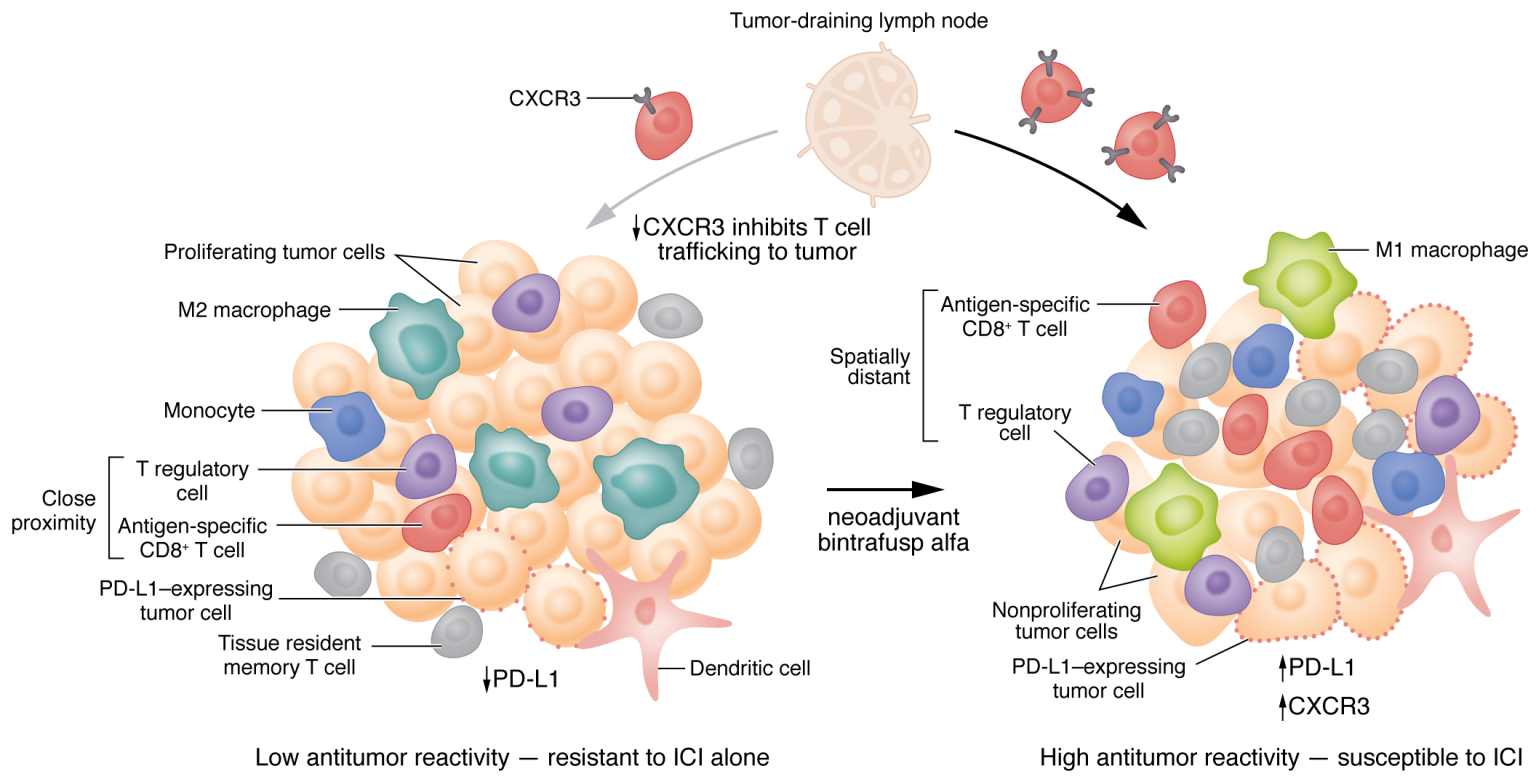
Model for how bintrafusp alfa may alter effector and suppressive elements in the tumor microenvironment to influence the response to immunotherapy. Tumors with low antitumor reactivity are characterized by interferon (IFN) production, decreased PD-L1 expression, a paucity of TILs or tissue resident memory T cells, an immunosuppressive (M2) monocytic phenotype, and a high number of Tregs and PD-L1-expressing cells in close proximity to CD8^+^ T cells. Tumors with high antitumor reactivity, which can be susceptible to ICI, are characterized by (a) cytokine and growth factor signals that can drive dendritic cell recruitment and maturation, (b) a pro-inflammatory monocytic (M1) phenotype, (c) robust antigen-specific CD8^+^ T cell and Trm infiltration, (d) chemokine binding that recruits activated effector T cells from the lymph nodes, (e) upregulation of PD-L1, and (f) few, if any, Tregs or PD-L1-expressing cells in close proximity to CD8^+^ T cells. Redman et al. (8) showed that the PD-L1- and TGF-β-blocking agent bintrafusp alfa induced a systemic immune response characterized by expanded activated, antigen specific T cells. Bintrafusp alfa may also act via CXCR3 on CD8^+^ T cells to permit trafficking to the tumor.

HPV-unrelated head and neck squamous cell carcinoma (HPV-unrelated HNSCC) presents a unique opportunity and challenge for cancer immunotherapy. Although the Cancer Genome Atlas revealed that HNSCC has a relatively high number of genomic alterations, which, theoretically, provides the advantage of a greater number of tumor-associated antigens for the immune system to recognize, response to immune checkpoint inhibitors (ICI) in this disease is modest. Only 13–20% of patients with recurrent or metastatic HNSCC benefit from treatment with programmed death-1 inhibitors (PD-1i) alone (1,2) or in combination with cisplatin (3). Tumors evade immune surveillance through various mechanisms, including the production of immunosuppressive cytokines such as transforming growth factor β (TGF-β). Notably, TGF-β decreases CD8^+^ T cell proliferation and effector function, promotes activation and proliferation of suppressive Tregs, promotes alternative macrophage differentiation, decreases antigen presentation and effector function, and increases macrophage trafficking to the site of inflammation (4). TGF-β also suppresses chemokine receptor CXCR3 in CD8^+^ T cells, thereby limiting their trafficking to the tumor (5) (Figure 1). Previous studies have shown that HNSCCs are heavily infiltrated with Tregs. Moreover, close proximity of FoxP3^+^ Treg cells to CD8^+^ T cells induces a loss of function of cytotoxic T lymphocytes and predicts poor outcomes (6). Other than programmed death ligand-1 (PD-L1) expression — which is used in HNSCC to stratify immunotherapy with pembrolizumab monotherapy or in combination with chemotherapy — biomarkers such as CD8^+^ T cell infiltration, tumor mutational burden, or immune-gene expression profiling have been explored, but have yet to be validated in this disease (7).

## A bifunctional fusion protein

In this issue of the *JCI*, Redman et al. (8) describe the results of a phase 1b trial in patients with HPV-unrelated HNSCC, testing the neoadjuvant, presurgical administration of bintrafusp alfa, a bifunctional fusion protein that blocks PD-L1 and neutralizes TGF-β. They also present data from a series of eloquent correlative studies that interrogated the tumor microenvironment (TME) for changes in composition and architecture following neoadjuvant therapy. Primary tumors displayed pathologic tumor responses (pTR), defined as the percentage of viable tumor area within the tumor bed, ranging from 3% to 70%. Primary tumors displayed pathologic tumor responses (pTR), defined as the percentage of viable tumor area within the tumor bed, ranging from 3%–70%. Although there were no patients with a pathologic complete response (pCR, defined as no residual tumor), 5 of 14 patients definitively demonstrated at least a partial pathologic response, defined as a pTR of greater than 50%, yielding an overall primary pTR rate of 36%. These results compare favorably with neoadjuvant PD-1i monotherapy, where HPV-unrelated HNSCC pTR rates of greater than 50% range from 6% to 22%, with nivolumab treatment (9) or pembrolizumab treatment, respectively (10). Alterations in Treg cell infiltration and spatial distribution relative to proliferating CD8^+^ T cells, as well as the detection of neoepitope-specific tumor T cell responses, which correlated with pTR, were unrelated to genomic features or tumor antigenicity. Since pTR was associated with reduced pretreatment myeloid cell tumor infiltration, this study suggests that the myeloid component may be a substantial barrier to this therapy in HPV-unrelated HNSCC. Although the exact identity of effector T cells that respond to bintrafusp alfa are not known, there is emerging evidence that tissue resident memory (Trm) T cells may play an important role, which can be potentially expanded by immune checkpoint blockade in combination with TGF-β neutralization.

Recent studies have shown that subpopulations of neoantigen-reactive CD8^+^ and CD4^+^ tumor infiltrating lymphocytes (TILs) have a Trm phenotype and are highly prognostic for patients with HNSCC and other cancer types (11, 12). Furthermore, a unique subset of tumor-reactive cells, identified by coexpression of CD39, CD103, and CD8 can be expanded by PD-1i or anti-OX40 treatment in patients with cancer (13, 14). The report from Redman et al. supports recent studies performed on samples from patients with HNSCC enrolled in a neoadjuvant trial of ipilimumab-plus-nivolumab. These studies showed that, in responding patients, treatment-expanded tumor T cell clones recognized several antigens and induced a systemic immune response in which activated T cells enriched for both preexisting and emergent clonotypes, undetectable prior to surgery, were detectable(15). Furthermore, other studies using single-cell RNA sequencing and T cell receptor sequencing have shown that the responding T cells lie within Trm populations and have exhausted or dysfunctional phenotypes distinct from blood-emigrant bystanders and regulatory TILs (16). Therefore, one of the challenges to effective immunotherapy with ICI is that the preexisting neoantigen reactive Trm T cells likely to be most responsible for effector and memory function, appear to have limited reinvigoration capacity. A multi-pronged strategy will likely be required beyond the T cell synapse — the interface between the antigen presenting cell and the T cell at which tumor recognition occurs. To improve immunotherapy response rates, the strategy will not only need to enhance antigen specific CD8^+^ T cell function, but also reverse immunosuppression in the TME. Differences in TME content of CD8^+^ T cells (e.g., exhaustion states) and regulatory immune cells (e.g., Foxp3^+^ CD4^+^ Tregs and myeloid-derived suppressor cells) as well as differences in T cell trafficking to the tumor and tumor-intrinsic immune suppression all serve as potential mechanisms underlying the divergence in immune cell interactions and responses to immunotherapy operating in the TME of HNSCC that should be therapeutically targeted.

## Translational relevance

Locally advanced, HPV-unrelated HNSCC is associated with a high rate of therapeutic resistance following standard-of-care surgery and risk–adapted adjuvant radiation or chemoradiation. PD-1i and other ICIs are currently being tested in the definitive setting for the treatment of locally-advanced HNSCC. Unexpectedly, in JAVELIN, a large randomized phase 3 trial that compared adjuvant avelumab combined with standard chemoradiation to chemoradiation alone, avelumab failed to improve either disease-free survival (DFS) or overall survival in patients with locally-advanced HNSCC (17). However, this result may have been predicted by preclinical mouse studies, which demonstrated that neoadjuvant immunotherapy improves long-term survival and enhances antitumor immune responses compared with the same therapy administered in the adjuvant setting (18). Furthermore, Huang et al. (19), demonstrated that pCR or major pathologic response (MPR), less than 10% viable tumor cells, after a single dose of PD-1i was associated with improved DFS in patients with high-risk resectable stage III/IV melanoma. Pathologic responses were associated with accumulation of TILs, which, likewise, were associated with clinical benefits. The role of pathological response and its effect on clinical benefits was recently evaluated for 6 trials where neoadjuvant treatment was given to melanoma patients (*n =* 192) (20), in which the pCR rate translated to an overall survival benefit assessed at 2 years. In comparison to PD-1i alone, combination immunotherapy with neoadjuvant nivolumab and ipilimumab in patients with HNSCC appears to be more effective, resulting in 54% of treated patients being downstaged and an MPR rate of 14% (21). Although combination immunotherapy is a promising approach to enhance response rates, given the large number of biologic agents of various mechanisms, the number of combinations that can be tested far outstrips the resources available to test them.

To deepen pathologic responses and improve survival, future neoadjuvant combinations should test ICIs with other ICIs, costimulatory agonists, vaccines, chemotherapy, and/or radiation to increase the degree of tumor recognition by antigen-specific T cells and eliminate immunosuppressive mechanisms in the TME (Figure 1) (22). Cytokine secretion resulting from T cell recognition of their cognate antigen can result in differentiation of suppressive myeloid cells (i.e., M2 macrophages) into pro-inflammatory macrophages (M1) and dendritic cells, whereas chemokine secretion can promote recruitment of additional immune cells into the tumor. While T cell activation can generate immunosuppressive functions, such as upregulation of PD-L1 in nearby cells, PD-L1 in the TME is generally a good prognostic feature. Moreover, PD-L1 may make the tumor more responsive to checkpoint inhibition, possibly in combination with a myeloid-targeted therapy. In contrast, those tumors lacking preexisting T cell recognition also lack pro-inflammatory cytokines, therefore myeloid cells differentiate into T cell suppressive phenotypes (M2) with impaired tumor trafficking. In this case, ICIs are unlikely to benefit the patient without alternative interventions.

In contrast to the approach tested in JAVELIN, there has recently been a surge of interest in combining immunotherapy with hypofractionated stereotactic body radiation therapy (SBRT). In early stage lung cancer, neoadjuvant PD-1i combined with SBRT resulted in a 53% MPR, as compared with 6% for PD-1i alone (23). The proposed mechanisms of synergy observed with this approach include (a) the release of tumor antigens and damage-associated molecular patterns; (b) deletion of Tregs; (c) upregulation of antigen-processing machinery, MHC class I, and death receptors such as Fas and NKG2D; (d) cytokine and chemokine induction; and (e) enhanced immune cell trafficking (24). Relevant to the Redman et al. study, the addition of Galunisertib, a TGF-β type I receptor inhibitor, to diminish the immunosuppressive effects of TGF-β in the TME, improved pathologic response rates to chemoradiation in a neoadjuvant clinical trial in patients with locally advanced rectal cancer (25). Changes in CXCR3^+^ CD8^+^ T cell tumor trafficking and activation status that correlated with response were suggested as possible mechanisms for the observed increase in clinical activity (Figure 1). In another study of neoadjuvant immunoradiotherapy testing SBRT-plus-nivolumab in patients with locally advanced HNSCC, the combination resulted in a high rate of pCR and MPR (60% in HPV-unrelated patients), with clinical to pathologic downstaging in 90% of the patients enrolled (26).

Compared with adjuvant therapy, neoadjuvant, preoperative immunotherapy provides the opportunity to: (a) determine the on-treatment therapeutic response of an individual patient; (b) reduce the tumor burden prior to surgery and facilitate more limited surgery; (c) deintensify or eliminate the need for adjuvant therapy; and (d) study the effect of therapy on the TME and immune landscape. Time will tell whether pathologic response is an accurate surrogate for survival, but as demonstrated by Redman et al. (8), reversal of immunosuppression and determined pathologic responses to bintrafusp alfa were associated with neoepitope-specific tumor T cell responses, lower myeloid cell tumor infiltration in the pretreatment tumors, and better pathologic responses than observed with PD-1i alone. If validated in larger clinical data sets, the findings suggest that concurrent neoadjuvant inhibition of myeloid cell tumor trafficking or function may be a rational strategy to improve pathologic response and clinical outcome in patients with HPV-unrelated HNSCC.

## Version 1 09/15/2022

Electronic publication
